# Single-step acid-catalyzed synthesis of luminescent colloidal organosilica nanobeads

**DOI:** 10.1186/s40580-022-00303-z

**Published:** 2022-03-07

**Authors:** Phornsawat Baipaywad, Seong Vin Hong, Jong Bae Kim, Jangsun Hwang, Jonghoon Choi, Hansoo Park, Taejong Paik

**Affiliations:** 1 School of Integrative Engineering, Chung-Ang University, Seoul, 06974 Republic of Korea; 2 Biomedical Engineering Institute, Chiang Mai University, Chiang Mai, 50200 Thailand

**Keywords:** Luminescent materials, Organosilica, Bioimaging, Nanoparticle

## Abstract

We present a single-step, room-temperature synthesis of fluorescent organosilica nanobeads (FOS NBs). The FOS NBs were synthesized under aqueous conditions using (3-aminopropyl)triethoxysilane (APTES) as the silicon source in the presence of l-ascorbic acid (L-AA). In the APTES/L-AA/water ternary phase, the hydrolysis and condensation reaction of APTES occurred under acidic conditions to form spherical FOS NBs with an average diameter of 426.8 nm. FOS NBs exhibit excellent colloidal stability in aqueous media. The formation of FOS NBs was complete within a 10 min reaction time, which indicates potential for large-scale mass-production synthesis of luminescent colloidal NBs. The FOS NBs exhibited blue photoluminescence (PL) under UV excitation in the absence of an additional high temperature calcination process or with the incorporation of any fluorophores. This phenomenon is attributed to the presence of carbon-containing defects, which act as luminescent centers formed by the reaction between amino groups in the APTES and l-ascorbic acid reductant. Finally, the results of a cytotoxicity test and cellular uptake experiments revealed that the FOS NBs showed potential as optical contrast agents for bioimaging.

**Graphical Abstract:**

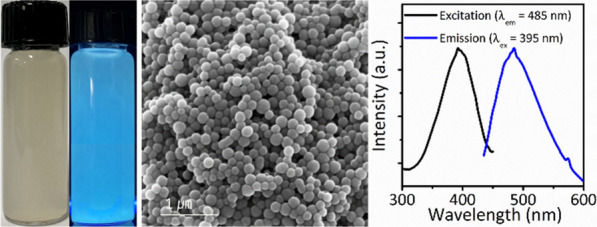

**Supplementary Information:**

The online version contains supplementary material available at 10.1186/s40580-022-00303-z.

## Introduction

Silica (SiO_2_) or organosilica (O–SiO_2_) nanobeads (NBs) have been extensively investigated for applications in biomedical applications and clinical medicine [1–5] including bioimaging [6, 7], drug delivery [8–12], and bioanalytical essays [13] owing to the non-toxicity, environmental friendliness, and low cost [14, 15]. In addition, owing to the large surface area of nano-sized SiO_2_ NBs, their surfaces can be modified with functional moieties to enable their application in imaging and delivery. For bioimaging applications, SiO_2_ NBs should possess luminescent properties to enable the tailoring of the presence and location of NBs in biological environments. Because SiO_2_ and O–SiO_2_ NBs do not exhibit luminescence, fluorophores such as organic dyes [16, 17], lanthanide ions [18, 19], and quantum dots (QDs) [20, 21] are usually incorporated into the matrix of SiO_2_ to endow it with luminescence properties. However, the incorporation of fluorophores into SiO_2_ NBs often involves multiple synthetic and extensive purification processes. In addition, some fluorophores are relatively expensive or contain toxic elements (e.g., cadmium in QDs). Therefore, it is important to develop a simple and inexpensive process to obtain luminescent SiO_2_ NBs to enable its mass production and large-scale applications in industrial applications.

Recently, the colloidal synthesis of label-free luminescent Si-based nanoparticles (NPs) has been reported for the application of Si-based NPs in bioimaging [22–24] and solar spectral converters [25]. The colloidal synthesis involves the synthesis of luminescence crystalline Si NPs via the thermal decomposition of hydrogen silsesquioxane, followed by the chemical etching of SiO_2_ to obtain H-terminated Si NPs [26]. Yang et al. reported a solution route for the synthesis of alkyl terminated Si NPs by the reaction between SiCl_4_ and Mg_2_Si in solvent [18]. The as-synthesized Si NPs exhibit characteristic blue-to-red luminescence properties depending on the size of Si NPs. Several studies have reported the synthesis of luminescence SiO_2_ or O–SiO_2_ NBs [27, 28]. For example, Jakob et al. reported a synthetic procedure for monodispersed luminescent O–SiO_2_ sphere via a modified Stöber process using (3-aminopropyl)trimethoxysilane (APTMS), followed by high-temperature calcination [29]. In addition, Chen et al. reported the synthesis of bright green emitting O–SiO_2_ NBs via the hydrothermal reaction between rose bengal and silane molecules [30]. Luminescent Si-based NBs show potential in biomedical applications owing to their biocompatibility and the tunability of their PL wavelength from ultraviolet to near infrared [31, 32]. However, the previously reported synthesis methods for luminescent Si-based NBs require a high-temperature calcination process or chemical etching with toxic chemicals, which affects the mass production process of Si-based NBs. In addition, high-temperature calcination often induces the aggregation of NBs, which may lead to the reduction of their dispersibility in aqueous media. Therefore, it is important to develop a simple, low-temperature method for synthesizing luminescent, photo-stable Si-based NBs.

Herein, we report the single-step, acid-catalyzed synthesis of FOS NBs at room temperature in the aqueous phase. The FOS NBs were synthesized in a APTES/L-AA/deionized (DI) water ternary phase. Acid-catalyzed hydrolysis and condensation of APTES occurred to form spherical FOS NBs. Thus far, most of the reported aqueous syntheses of Si-based luminescent NPs using organosilane and organic reductants have produced Si-based NPs with diameters less than 10 nm [32–34]. However, in our study, we observed that spherical NBs of diameter ~ 430 nm are actually formed as the main product after the room-temperature reaction (25 °C) progressed for 10 min, which exhibited blue PL properties. In addition, the FOS NBs exhibited excellent colloidal stability in aqueous medium. Furthermore, the luminescent FOS NBs exhibited blue PL properties at 485 nm of maximum under UV excitation. The cytotoxicity test and cellular uptake experiments were performed using the FOS NBs, and the results revealed that the FOS NCs showed potential as an optical contrast agent for long-term cell imaging owing to heir negligible cytotoxicity and biocompatibility.

## Methods and materials

### Chemicals

APTES (99%), APTMS (99%), L-AA, and ethanol (EtOH, 98%) were purchased from Sigma-Aldrich. All the chemicals were used as received without additional purification. The DI water used in all the reactions and for cleaning the glassware was purified to a resistance of 18 MΩ and filtered through a 0.22-µm membrane to remove any impurities.

### Synthesis of FOS NBs

To synthesize the FOS NBs, first, 0.149 g (0.85 mmol) of L-AA was dispersed in 8 mL of argon-saturated DI water solution, and the mixture was stirred at 25 °C for 10 min. Next, 2 mL (0.95 mmol) of APTES was added to the above mixture, and the mixture was stirred for 10 min. Subsequently, the supernatant was discarded and extra ethanol was added into the reaction flask, after which the mixture was sonicated. The FOS NBs were collected via centrifugation and washed with ethanol three times by centrifugation.

### Characterizations

The scanning electron microscopy (SEM) images of the samples were obtained using a Carl Zeiss SIGMA operated at an accelerating voltage of 5 keV. Electron diffraction spectroscopy (EDS) was performed using a scanning electron microscope equipped with an energy-dispersive X-ray spectrometer. Transmission electron microscopy (TEM) images were obtained using a JEOL JEM-2100 instrument operating at 200 kV. The TEM samples were prepared by dispersing the as-synthesized FOS NBs in ethyl alcohol, after which the mixture was dropped on a carbon-coated Cu grid at room temperature. The size distribution and surface charge of the nanoparticles were measured using a NANO ZS laser particle analyzer system (Malvern instruments Ltd., UK). A He–Ne laser was used as the light source at an incident wavelength of 633 nm and measurement angle of 173°. ζ-potential measurements were carried out using highly diluted colloidal dispersions of the FOS NBs and were measured at 25 °C. The Fourier-transform infrared (FTIR) spectra of the samples were recorded on a Bruker FTIR Spectrometer Model: ALPHA II. The spectra were measured from 500 to 4000 cm^−1^ in an attenuated total reflectance (ATR) mode. Powder X-ray diffraction (XRD) patterns were obtained on a Bruker AXS/new D8 Advance X-ray diffractometer using a Cu Kα radiation source in the scan range (2θ) from 10 to 60°. X-ray photoelectron spectroscopy (XPS) profiles were obtained on a Thermo Scientific K-Alpha using the EX06 ion source. The UV–visible (UV–vis) absorption spectra of the samples were recorded using a JASCO V-770 UV–vis/NIR spectrometer. The PL spectra were obtained using an Edinburgh FS5 spectrofluorometer. The absolute quantum yield (QY) was measured using an integrating sphere.

### CCK-8 assay of the cell viability

To investigate the viability of human adipose-derived stem cells (hASCs) to the as-synthesized FOS NBs, cell counting Kit-8 (CCK-8, Dojindo) was employed. hASCs isolated from a primary culture of a patient’s tissue were obtained from the CHA medical center. The cultured hASCs were seeded onto 96-well plates (4 × 10^4^ cells per well) in a low-glucose Dulbecco’s modified Eagle medium (DMEM) supplemented with 10% (v/v) fetal bovine serum and 1% (v/v) antibiotic–antimycotic solution. The cells were incubated for 24 h under standard cell culture conditions (37 °C, 5% CO_2_, and 95% humidity). Subsequently, the untreated control (negative control), tritonTM X-100 treated control (positive control), and 50, 100, and 500 µg mL^−1^ of the FOS NBs were dispersed in DMEM and added to the cells, after which the cells were cultured for 24 and 48 h. The results were quantified as a function of the negative control, which was considered to have 100% viability. All the experiments were performed in triplicate. The cell viability was measured according to the cytotoxicity protocol. Briefly, the samples within cultured media in the 96-well plates were removed and washed with Dulbecco’s phosphate-buffered saline (DPBS). Subsequently, 5% (v/v) of CCK-8 solution in DMEM was added to the cells, and the cells were incubated for 4 h. The absorbance at 450 nm was measured using a microplate reader (SynergyTM H1, BioTek instruments Inc.).

### Cell imaging experiments

Briefly, hASCs were seeded onto cell culture slides in eight-well plates at 1 × 10^5^ cells per well and cultured for 24 h. After the attachment period, the cells were incubated with the FOS NBs for 24 h in the medium. After removing the medium, the cells were fixed using 4% paraformaldehyde for 1 h, after which 1% tritonTM X-100 was added for 1 h, and the wells were washed thrice with DPBS to remove the unbound compounds. Subsequently, the cell nuclei were stained with a nucleus-selective dye (DAPI) for 30 s and washed thrice with DPBS. The cell was observed by confocal laser scanning microscopy (LSM880, Carl Zeiss Microscopy, United States).

### Statistical analysis

The data are presented as the mean ± standard deviation. The resulting values from an experiment were compared by the two-way analysis of variance (ANOVA) and multiple comparisons between the control group and sample groups were performed. Statistical significance was set at a level of P < 0.0001.

## Results and discussion

**Fig. 1:**
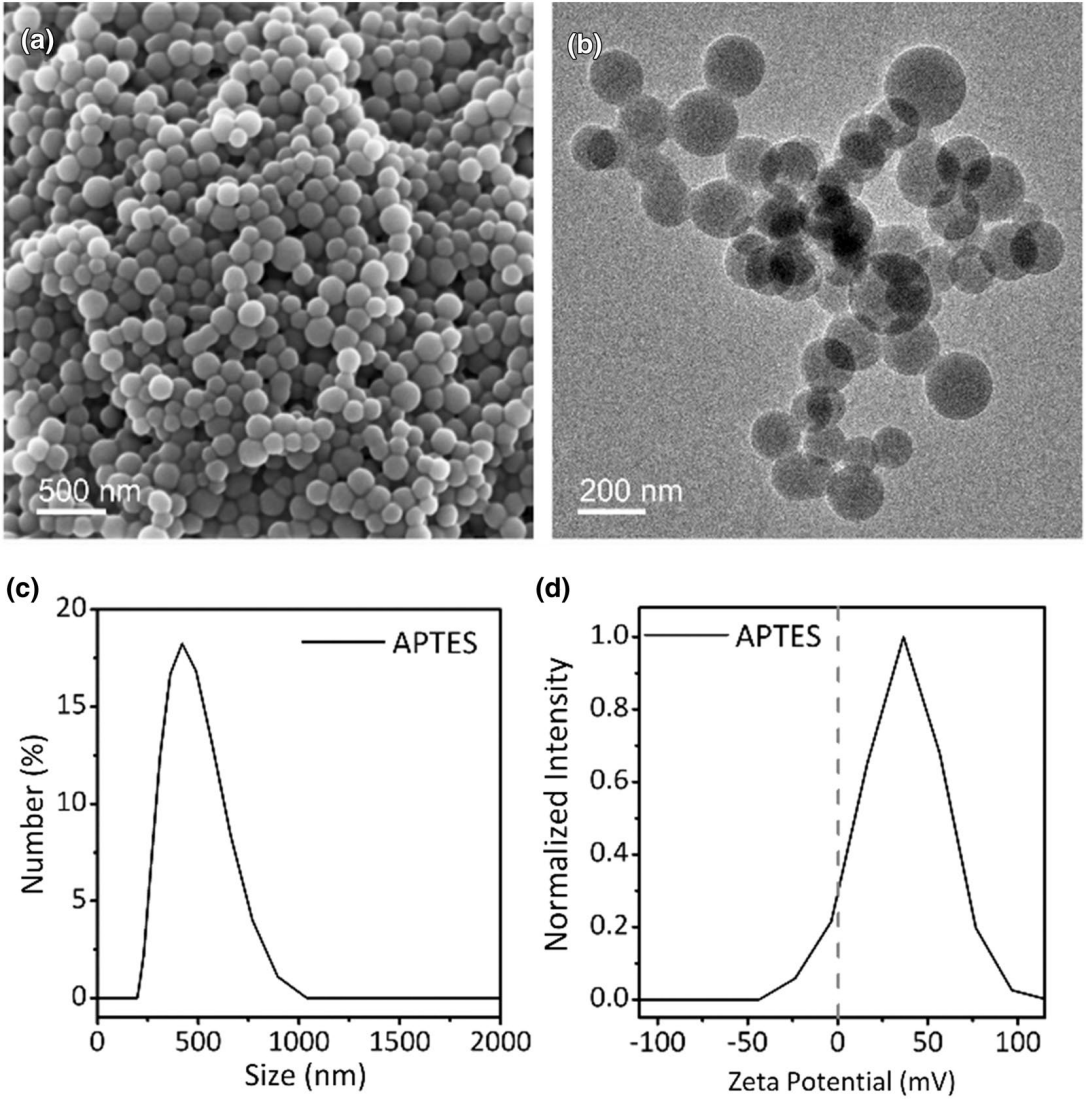
**a** SEM and **b** TEM images of the FOS NBs synthesized using APTES precursors. c) DLS and **d** ζ-potential measurements of the as-synthesized FOS NBs

FOS NBs were synthesized via a single-step, room-temperature method under an air atmosphere using aminopropyl silane as the organosilane source in the presence of L-AA and DI water. First, L-AA was dispersed in DI water, after which the brownish organosilane was added to the reaction solution to induce the formation of the FOS NBs. After the addition of the organosilane precursors, the color of the solution turned into a reddish color after 5 min. Ten minutes after the reaction initiation, the FOS NBs were collected via removing supernatant and adding extra ethanol into the reactors followed by centrifugation to obtain FOS NBs. Figure 1 shows the SEM images of the FOS NBs synthesized using APTES precursors. As shown in Fig. 1a, b, the FOS NBs exhibit spherical morphologies. The TEM images also reveal that the FOS NBs consist of amorphous structures without crystalline fringe patterns. Figure 1c shows the size histogram of the FOS NBs obtained via dynamic light scattering (DLS) measurement. The average size of the FOS NBs was approximately 426.8 nm with a narrow size distribution. In addition, although the FOS NBs were synthesized using different types of organosilane monomers (Additional file 1: Fig. S1), the type of organosilane monomer used for the reaction does not significantly influence on the size of the FOS NBs; however, the polydispersity index of the FOS NBs synthesized with APTES was smaller than those synthesized with APTMS. In addition, the size and size distribution were not significantly varied by changing the reaction temperature (25 °C/40 °C) and reaction time (20 min/24 h/48 h). Furthermore, although the surface of SiO_2_ NBs is negatively charged owing to the silanol group on the surface, ζ-potential measurements revealed the presence of positive charges on the surface of the FOS NBs (Fig. 1d). The positive charges on the surface of the FOS NBs could be attributed to the presence of an amine group in the organosilane monomers, which formed positively charged ammonium moiety on the surface of the FOS NBs. The ammonium groups on the surfaces of the FOS NBs enabled the colloidal stability of the FOS NBs in DI water owing to the electrostatic interaction between the ammonium groups. Therefore, the as-synthesized FOS NBs exhibited excellent colloidal stability without precipitation in DI water.

**Fig. 2:**
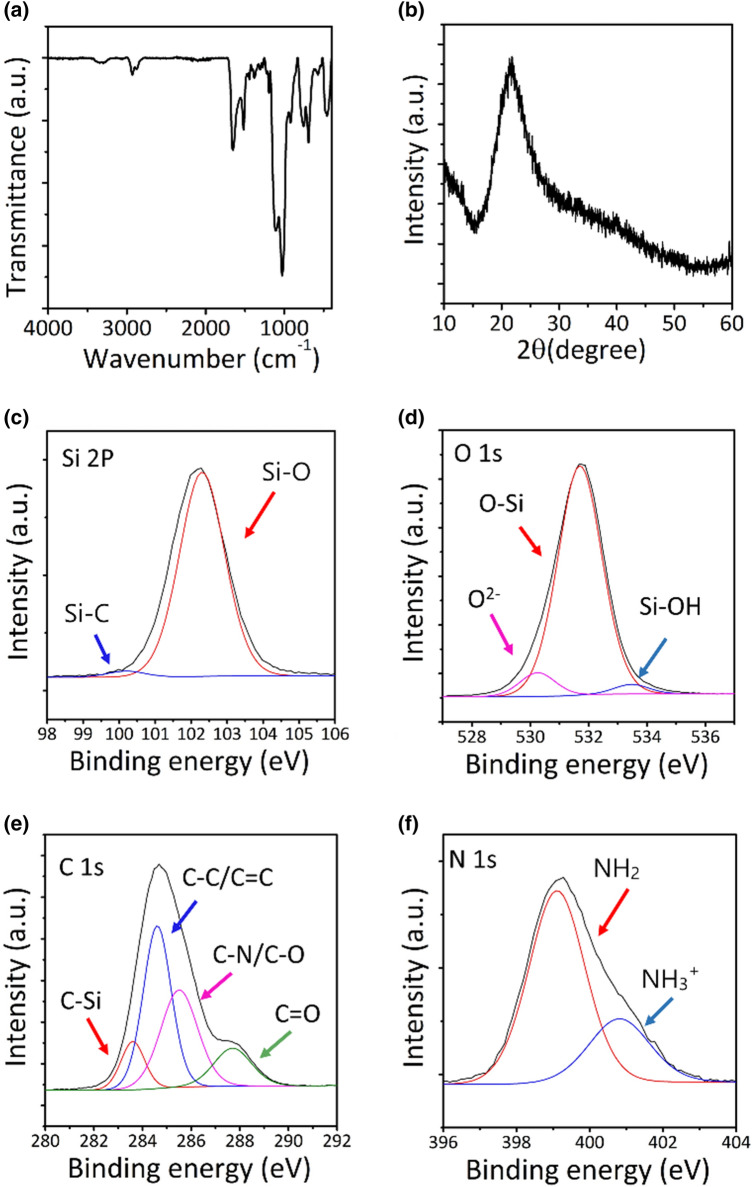
**a** FTIR spectra and **b** XRD pattern of the FOS NBs. High resolution XPS spectra of **c** Si 2p, **d** O 1 s, **e** C 1 s, and **f** N 1 s, and peaks of the luminescent FOS NBs

The chemical structure of the FOS NBs was characterized using FTIR measurements. Figure 2a shows the FTIR spectra of the FOS NBs. A sharp and strong peak was observed at 1031 cm^−1^, which is attributed to the characteristic Si–O stretching. In addition, a sharp absorbance peak was observed at 1652 cm^−1^, which corresponded to the asymmetric –NH_3_^+^ deformation mode [35], indicating the presence of amine group in the NBs. Furthermore, two additional peaks were observed at 1516 cm^−1^ (N–H bending) and 3340 cm^−1^ (N–H stretching), indicating the presence of –NH_2_ groups in the FOS NBs. The absorbance peaks from 690 to 755 cm^−1^ indicated that free water was adsorbed on the surface of the NBs via hydrogen bonding [36]. Furthermore, the peak observed at 2931 cm^−1^ corresponded to the C–H vibration of aminopropyl groups. In addition, the shoulder was observed at 919 cm^−1^, which corresponded to the existence of silanol groups (Si–OH) in the FOS NBs. These results indicate that the FOS NBs mainly consisted of aminopropyl silane groups. In addition, a broad peak with a Bragg angle at 2θ = 21.7° was observed in the XRD pattern, confirming the amorphous structure of the FOS NBs (Fig. 2b).

For the elemental analysis of the as-synthesized FOS NBs, XPS measurement was performed on the FOS NBs. Five peaks were observed in the full-range XPS spectra (Additional file 1: Fig. S2) of the FOS NBs at 102.5, 152.7, 285.9, 400.4, and 532.4 eV, which were attributed to Si 2p, Si 2s, C 1s, N 1s, and O 1s, respectively. In addition, the high-resolution XPS spectrum of Si 2p (Fig. 2c) revealed that the silicon existed in two different chemical environments originating from Si–O (102.3 eV) and Si–C (102.2 eV) [37], which corresponds to the aminopropyl silane group. The three fitted peaks at 533.4, 532.2, and 530.2 eV in the O 1s spectrum (Fig. 2d) were assigned to Si–O, Si–OH, and O^2−^ groups, respectively. In addition, four peaks were observed in the C 1s spectrum (Fig. 2e) at 287.7, 285.5, 284.6, and 283.6 eV, which could be attributed to C=O, C–N/C–O, C–C/C=C, and C–Si [30], respectively, indicating the presence of aminopropyl group in the FOS NBs. Furthermore, the peaks at 400.81 and 399.1 eV in the N 1s spectrum (Fig. 2f) could be attributed to the presence of –NH_3_^+^ and –NH_2_ [38], indicating the presence of positively charged ammonium functional group in the FOS NBs, which is consistent with the corresponding FTIR spectrum. The structural analysis of the FOS NBs by FTIR, XRD, and XPS confirmed that the as-synthesized FOS NBs were amorphous organosilica with positively charged ammonium functional groups on their surfaces.

The formation of FOS NBs occurred via the acid-catalyzed reaction of silane monomers in the silane/acid/DI water ternary phase [39, 40]. Synthesis of spherical SiO_2_ NBs with a diameter range of hundreds of nanometers to micrometers has been reported for various types of organic (e.g., acetic, tartaric) or inorganic (hydrochloric, nitric) acids. It is reported that the particle size was controlled by varying the relative molar ratio of the silane monomers and acids [41–43]. In our system, L-AA induces hydrolysis and condensation reactions of silane monomers to form FOS NBs with a diameter of several hundred nanometers. After a 10 min reaction time, the precipitated sols sank to the bottom of the flask, leaving a clear, reddish supernatant liquid. These precipitated sols may have been the heavy liquid phases of the reaction intermediates, for example, polysilicic acid [42]. There were no FOS NBs present in the supernatant after centrifugation, therefore, the supernatant was discarded, leaving the reaction precipitates in the reaction flask. After adding an excess amount of EtOH to the flask, the solution became cloudy, indicating the separation and dispersion of FOS NBs in EtOH.

**Fig. 3:**
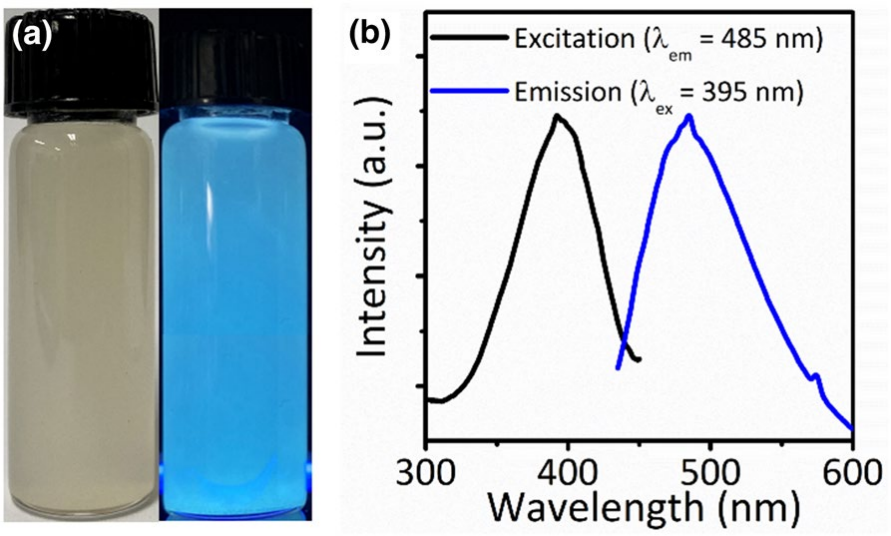
**a** The photograph of FOS NBs dispersed in DI water under white light (left) and UV irradiation (right), **b** fluorescent excitation/emission spectra

L-AA contributed not only to the reaction of silane monomers but also to the incorporation of luminescent properties into the FOS NBs. Figure 3a shows the photograph of the colloidal dispersion of the FOS NBs under white light and UV irradiation of 365 nm. The FOS NBs formed a milky dispersion in DI water, which could be attributed to the scattering by several hundred nanosized NBs. In addition, the FOS NBs exhibited blue PL under UV irradiation without high-temperature calcination. Figure 3b shows the excitation and PL spectra of the FOS NBs. The maximum PL emission peak of the FOS NBs was observed at 485 nm (Fig. 3b) and the PL spectra was independent of the excitation wavelength (Additional file 1: Fig. S3). In addition, the PL spectra of the FOS NBs synthesized for 10 min, 12 h, and 24 h were measured. The PL and PL excitation (PLE) spectra of all the samples were almost identical properties of the FOS NBs (Additional file 1: Fig. S4). The absolute photoluminescence quantum yield (PLQY) of the FOS NBs measured using an integrating sphere was approximately 2.4%. The long-term stability of PL properties under storage was observed under ambient conditions. The FOS NBs dispersed in DI water were stored for 30 d in an atmospheric environment, and their PL properties were investigated. The results revealed that long-term storage did not significantly deteriorate the PL properties of FOS NBs (Additional file 1: Fig. S5).

Thus far, several studies have reported the synthesis of Si-based NPs by the reaction between amine-containing organosilanes and organic reductants, such as L-AA and citric acid in aqueous medium [32–34, 44, 45]. Early literature argues that the luminescence properties of these Si-based NBs could be attributed to the presence of reduced crystalline Si owing to the reduction of the siloxane monomers by the organic reductants. However, the structure and origin of the luminescent properties of the Si-based NPs synthesized in aqueous medium are still unclear because nano-sized Si is susceptible to oxidation in aqueous medium [46, 47]. In this study, we confirmed that the FOS NBs were composed of the organosilica group. Furthermore, no strong evidence of the presence of zerovalent Si in the NBs was observed as corroborated by the FTIR, XPS, and XRD results. In addition, recent studies have reported that the products formed via the reaction of alkylamine and organic reductants exhibit similar luminescence properties as Si-based NBs [46]. Therefore, the results of this study suggest that the silane groups were not reduced to zerovalent Si, but only acted as nano-sized organosilica templates, indicating that the luminescence properties of the FOS NBs may have originated from the reaction between amine group and L-AA. This could be related to the formation of luminescence carbon-containing particles, such as carbon dots [48–51]. Although previous studies have reported that luminescent carbon-containing particles are formed under high-temperature thermal decomposition conditions that induce carbonization, our result revealed that luminescent particles can also be formed under room-temperature conditions within a short reaction time (10 min).

In addition, several literatures have reported that the reaction between organosilane and organic reductants induces the formation of nanoparticles of less than tens of nanometers in size [32–34, 44, 45]. However, we observed that the reaction products were mainly NBs of several hundreds of nanometers in size. As previously discussed, the color of the reaction solution changed to a reddish color. After 10 min of the reaction, the solution retained its red color, as shown in Additional file 1: Fig. S6a; however, white precipitates were observed at the bottom of the reaction flask. Subsequently, the supernatants were selectively discarded, and extra ethanol was added to collect the white precipitates in the flask. After adding ethanol, the precipitates were redispersed in ethanol to form a milky solution (Additional file 1: Fig. S6b), which is the obtained colloidal dispersion of FOS NBs in ethanol. The PL properties of the supernatant and precipitates were measured under UV irradiation of 365 nm. The results revealed that the luminescence properties were mainly observed in the precipitates (Additional file 1: Fig. S7). This result confirmed that the PL properties, which originated from the carbon-based luminescent centers, is attributed to the organosilica NBs.

**Fig. 4:**
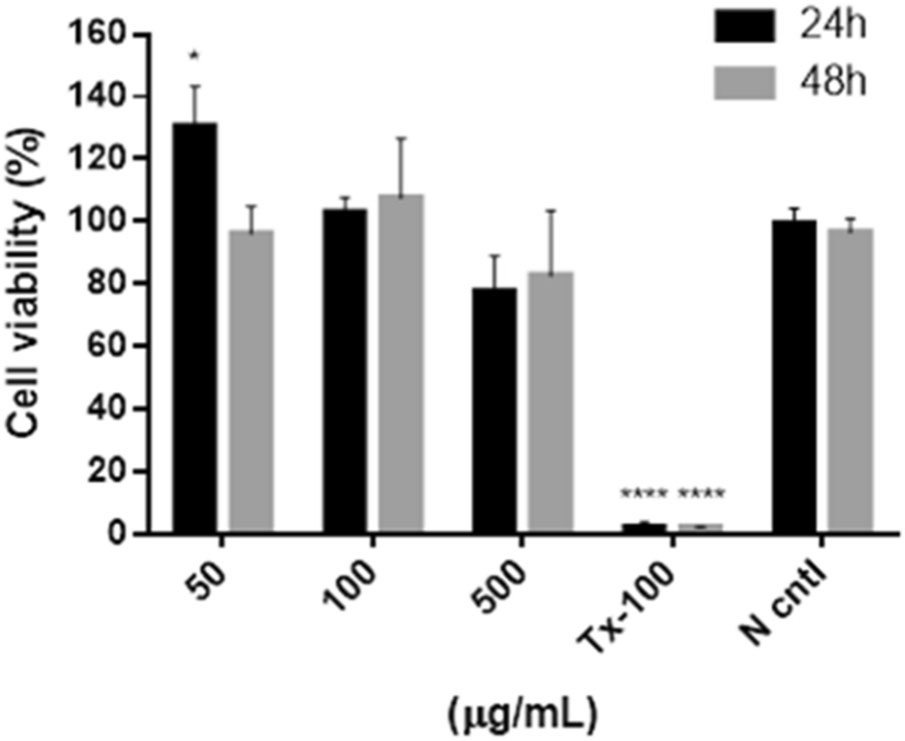
Cell viability of the hASCs cells determined by the CCK-8 assay. Materials were incubated with cells for 24 and 72 h with different concentrations of FOS NBs seeding to cells

To investigate the potential utilization of the FOS NBs in bioimaging application, the cytotoxicity of the FOS NBs was investigated by CCK-8 assay (Fig. 4). The cytotoxicity of the FOS NBs was measured during 24 h and 48 h incubation periods using triton X-100 as the positive control. Triton X-100 exhibited complete toxicity to the hASCs in terms of viability loss. The FOS NBs were injected into the hASCs at concentrations of 50, 100, and 500 µg mL^−1^. At a concentration of 500 µg mL^−1^, the FOS NBs exhibited slight cytotoxicity toward the hASCs, which may be due to the presence of positively charged ammonium groups on their surface known to induce toxicity. This is accounted for more ability of positively charged NBs easily enter to cells due to the electrostatic attraction between the negatively charged cell membrane glycoproteins and positively charged NBs and being slightly toxic [52]. However, the cells preserved over 80% viability, demonstrating the good biocompatibility of the FOS NBs.

**Fig. 5:**
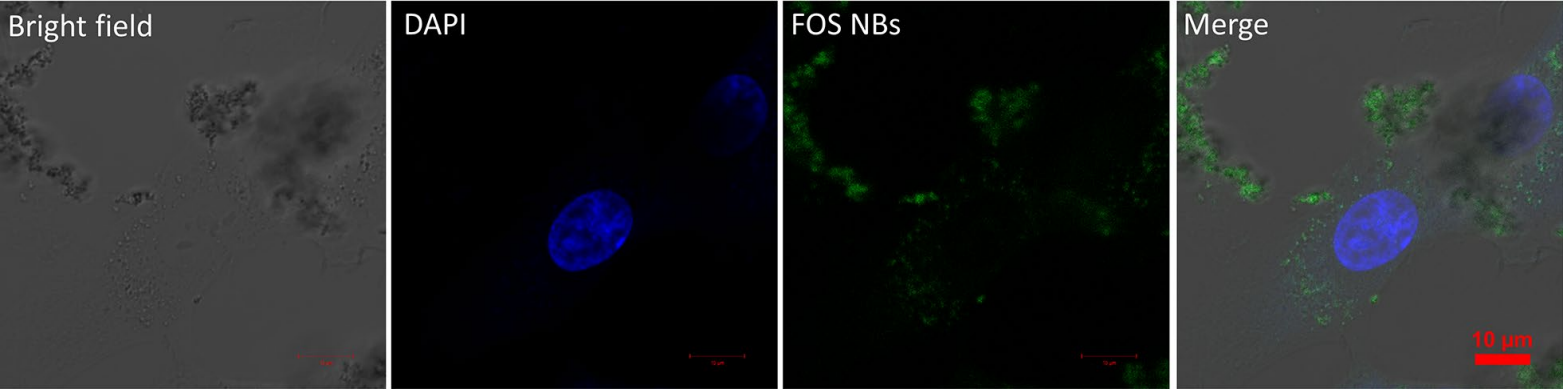
Fluorescence microscopy images of the hASCs with FOS NBs and DAPI

Finally, we investigated the potential of FOS NBs for cellular imaging applications owing to their biocompatibility and luminescence properties. The cellular uptake of FOS NBs into the hASCs was evaluated by examining the intrinsic fluorescence of the NPs by confocal laser scanning microscope (LSM). The hASCs were incubated with the FOS NBs (50 µg/mL) for 24 h, and cell nuclei were selectively stained with a nucleus-selective dye (DAPI) to located cell location. As shown in Fig. 5, the greenish emission is observed in label-free FOS NBs. This is attributed to the fact that FOS NBs exhibit the relatively broad emission from blue to green wavelength and blue emission from FOS NBs were removed from blue filter of confocal LSM. It is observed that FOS NPs were effectively internalized into cells and selectively distributed in the lysosomes around nuclei stained using DAPI. In addition, no fluorescence signal of FOS NBs was observed in the nuclei, indicating that the NPs could not penetrate the nuclear membrane. This result indicates the potential of the label-free FOS NBs for effective drug delivery applications in future studies.

## Conclusions

In summary, we successfully synthesized luminescent FOS NBs via the acid-catalyzed hydrolysis and condensation reaction of APTES in the presence of L-AA in a single-step, room-temperature reaction. The as-synthesized FOS NBs exhibited a spherical morphology with an average diameter of approximately 426.8 nm. In addition, the FOS NBs exhibited excellent colloidal stability in aqueous media owing to the electrostatic interaction between the positively charged ammonium groups on their surfaces. Furthermore, the XRD, XPS, and FTIR characterizations confirmed the amorphous organosilica structure of the FOS NBs. The FOS NBs exhibited blue PL properties under UV excitation, which was attributed to the presence of carbon-containing defects due to the reaction between the amine functional groups and the L-AA reductant. The cytotoxicity test revealed the biocompatibility of the FOS NBs, and cellular uptake experiments revealed that the FOS NBs penetrated the lysosomes. These results highlight the potential of FOS NBs as optical contrast agents for bioimaging.

## Supplementary Information

**Additional file 1.** Additional TEM images, XPS data, and PL results.

## Data Availability

The authors have no data to share since all data are shown in the submitted manuscript.

## Abbreviations

FOS NBs: Fluorescent organosilica nanobeads
APTES: (3-Aminopropyl)triethoxysilane
L-AA: l-Ascorbic acid
O–SiO_2_: Organosilica
hASCs: Human adipose-derived stem cells
CCK-8: Cell counting Kit-8
PLQY: Photoluminescence quantum yield
