# Correction: Single-step acid-catalyzed synthesis of luminescent colloidal organosilica nanobeads

**DOI:** 10.1186/s40580-022-00347-1

**Published:** 2022-12-09

**Authors:** Phornsawat Baipaywad, Seong Vin Hong, Jong Bae Kim, Jangsun Hwang, Jonghoon Choi, Hansoo Park, Taejong Paik

**Affiliations:** 1 School of Integrative Engineering, Chung-Ang University, Seoul, 06974 Republic of Korea; 2 Biomedical Engineering Institute, Chiang Mai University, Chiang Mai, 50200 Thailand

**Correction: Nano Convergence (2022) 9:12** 10.1186/s40580-022-00303-z

Following publication of the original article [1], the author noticed the error in Acknowledgement section. An incorrect grant number was cited in the published version. The grant acknowledged, “NRF-2019R1A4A1028700” should be “NRF-2022R1A4A2000776”. This has been corrected with this erratum.
