# Supplementary material for: Single-step acid-catalyzed synthesis of luminescent colloidal organosilica nanobeads
Source: Nano Converg. 2022 Mar 7;9:12. doi: 10.1186/s40580-022-00303-z (PMC8901841; doi:10.1186/s40580-022-00303-z)
Supplement: Supplementary file 1 — Additional file 1. Additional TEM images, XPS data, and PL results. [file 40580_2022_303_MOESM1_ESM.doc]

**Additional materials:**

**Single-Step, Acid-Catalyzed Synthesis of Luminescent Colloidal Organosilica Nanobeads**

Phornsawat Baipaywad^1,2^, Seong Vin Hong^1^, Jong Bae Kim^1^, Jangsun Hwang^1^, Jonghoon Choi^1^, Hansoo Park^1,*^, and Taejong Paik^1,*^

^1^ School of Integrative Engineering, Chung-Ang University, Seoul 156-756, Republic of Korea

^2^ Biomedical Engineering Institute, Chiang Mai University, Chiang Mai 50200, *Thailand*

^*^Corresponding authors: paiktae@cau.ac.kr (T. Paik), heyshoo@gmail.com (H. Park)

**
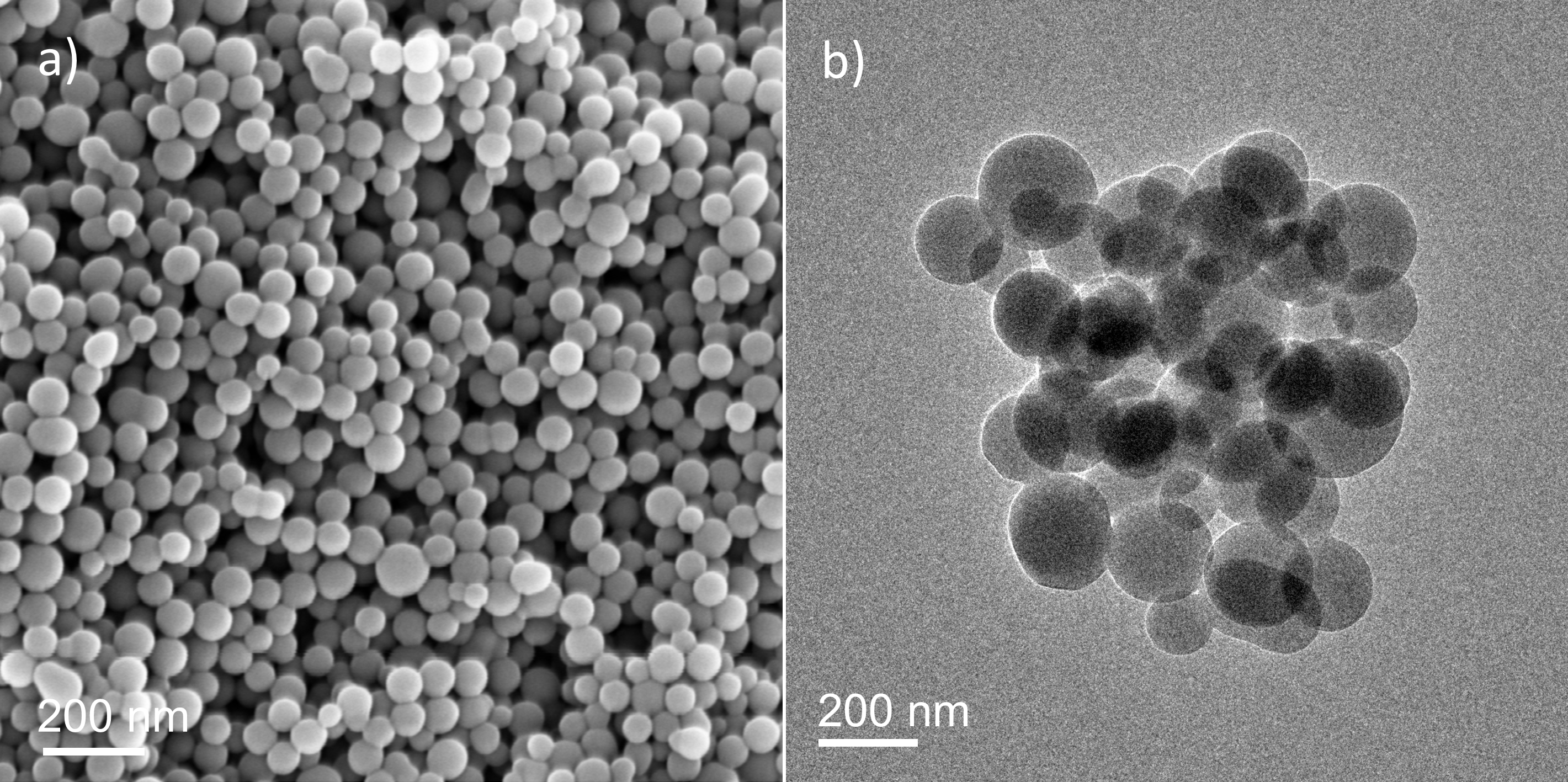
**

**Figure S1.** a) SEM and b) TEM images of FOS NBs synthesized using (3-aminopropyl)trimethoxysilane (APTMS) precursors.

**
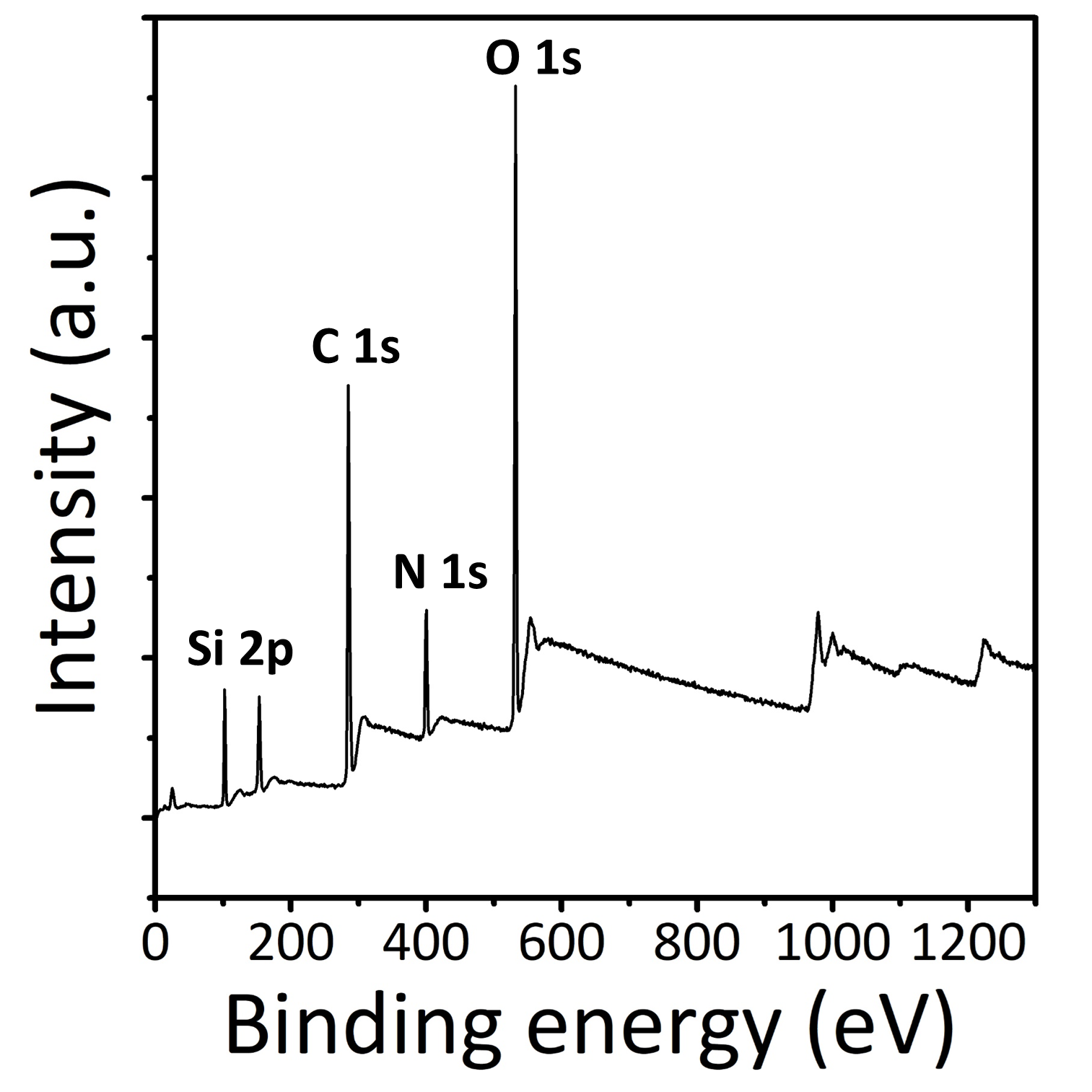
**

**Figure S2.** Full-range XPS spectra of the FOS NBs.


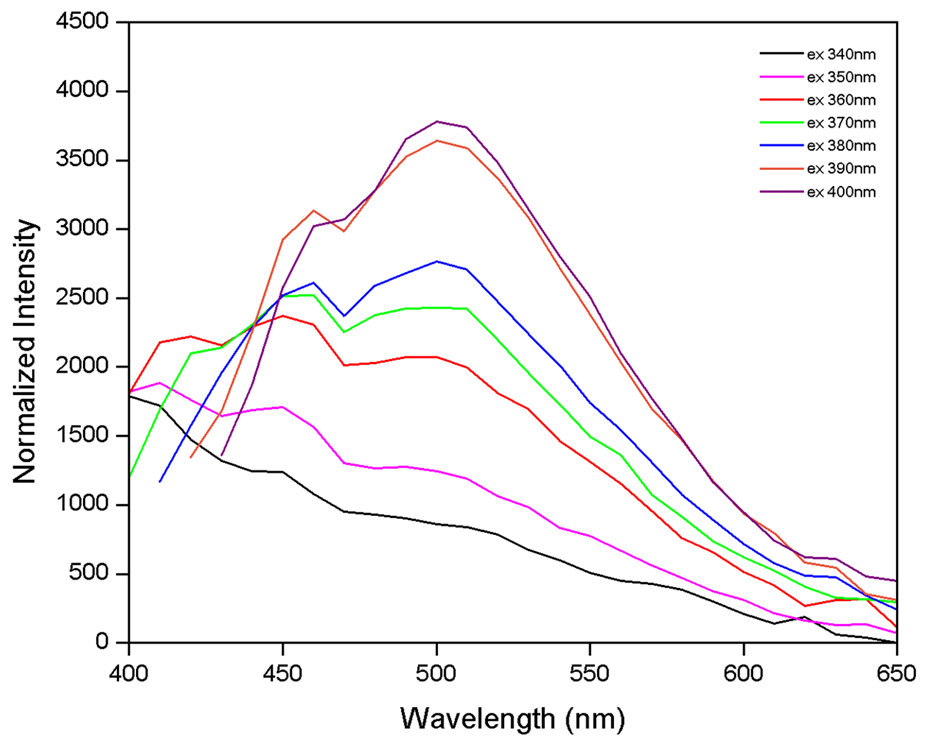


**Figure S3.** PL emission spectra of the luminescent FOS NBs at different excitation wavelengths.

**
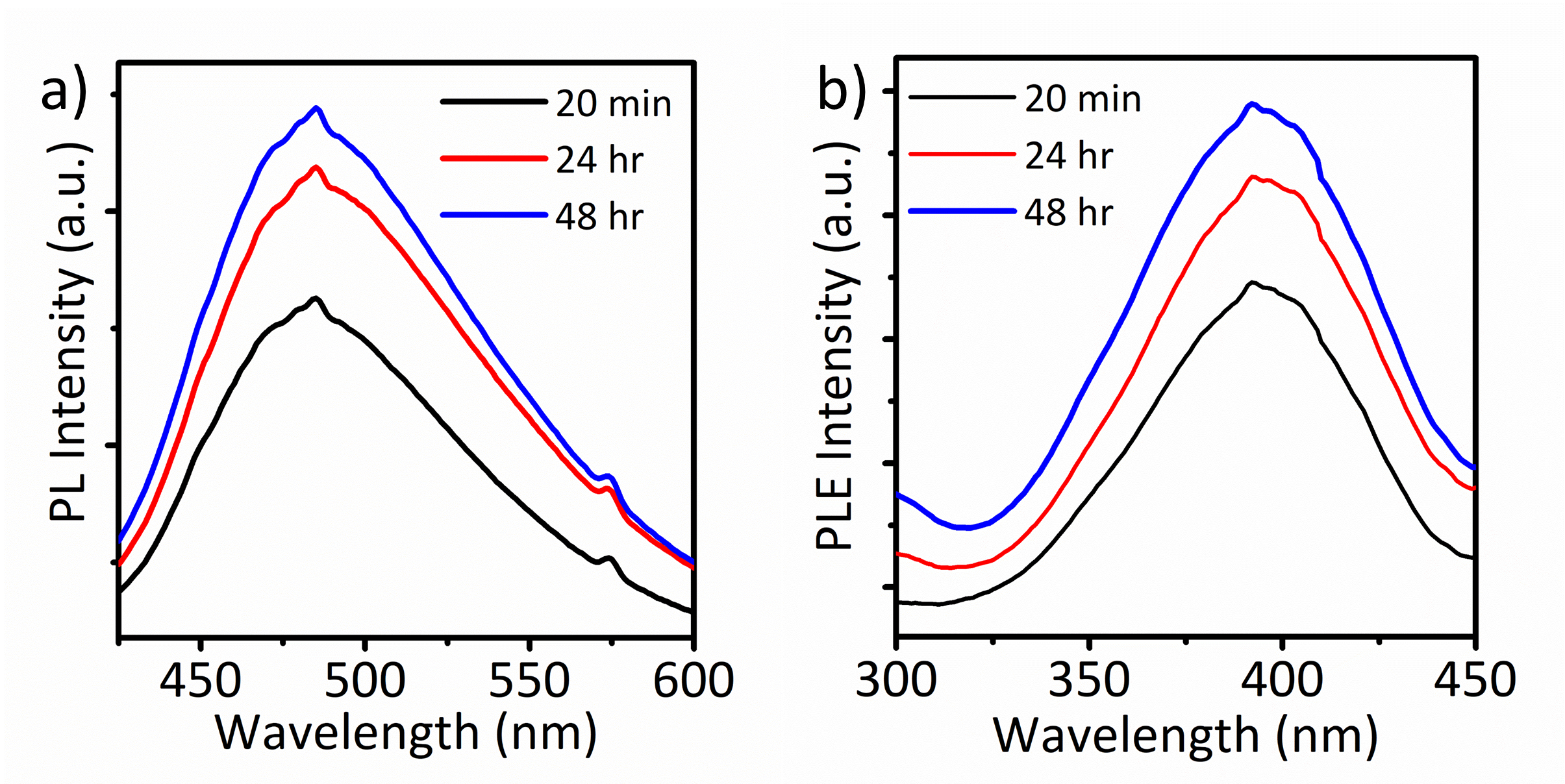
**

**Figure S4.** PL and PLE spectra of the FOS NBs synthesized over reaction times of 20 min, 12 h, and 24 h.


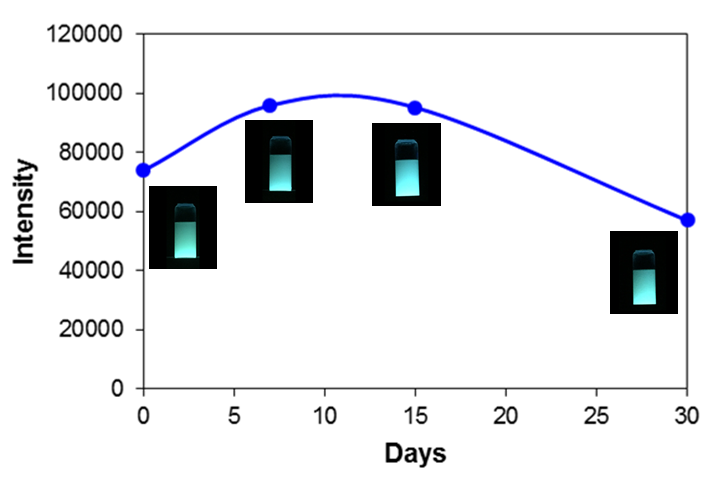


**Figure S5.** Stability and efficiency of the FOS NBs.


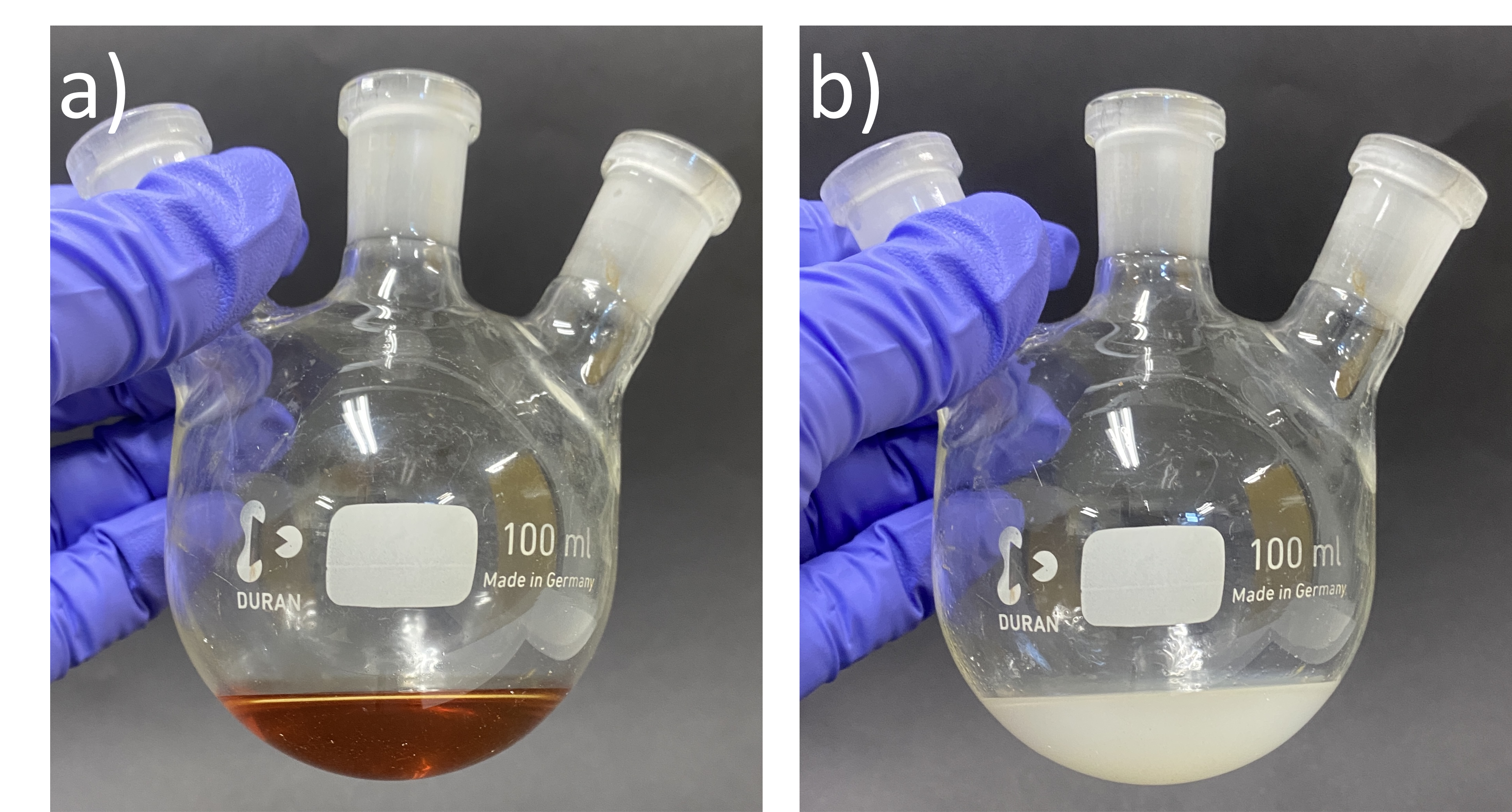


**Figure S6.** a) Supernatant exhibiting a red color after 20 min of reaction and b) collection of the precipitated FOS NBs to form a milky dispersion in ethanol.


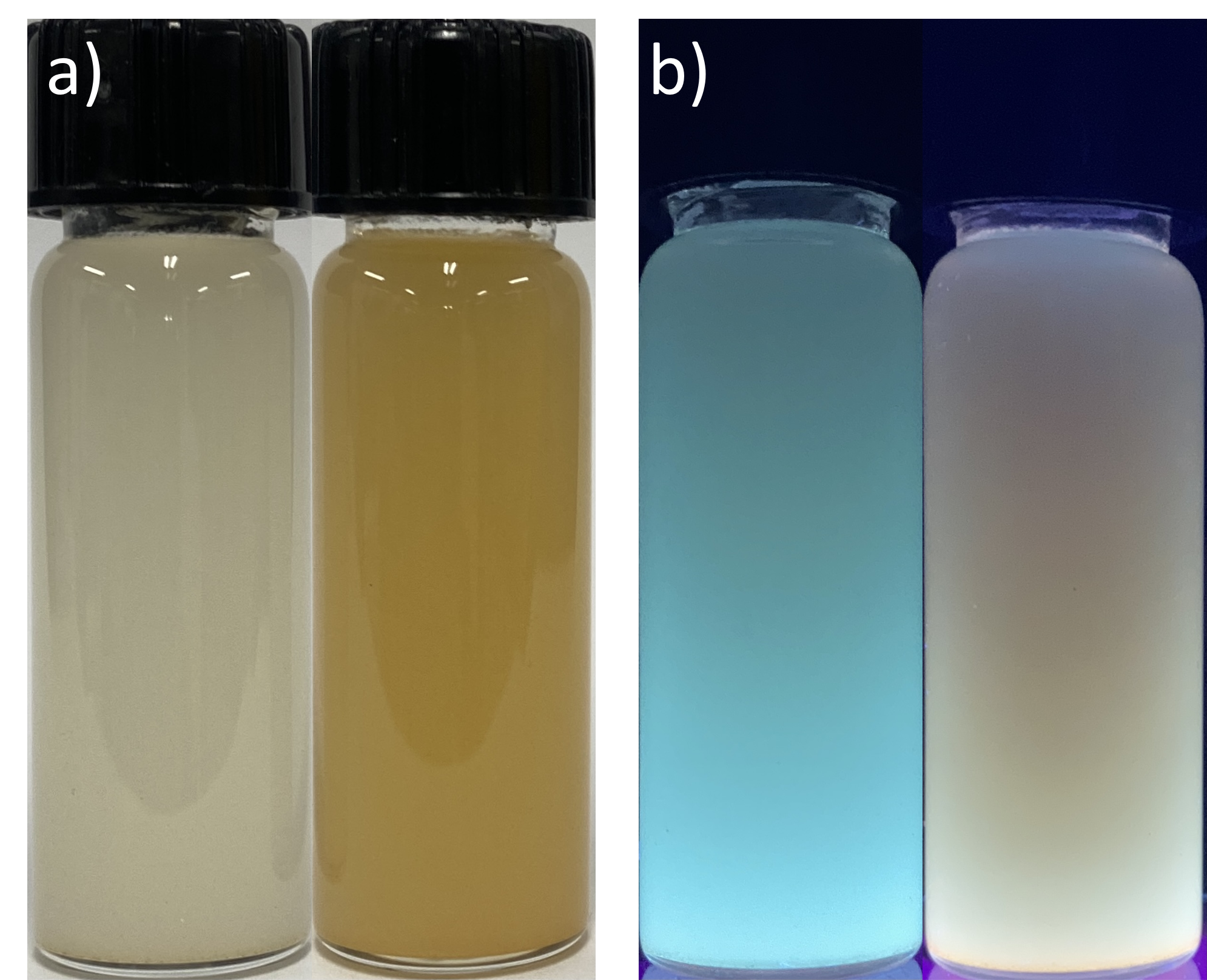


**Figure S7.** FOS NBs collected from the precipitates and dispersed in ethanol, and the supernatant a) under white light and b) in a dark room with UV.
